# Patient-derived tau and amyloid-β facilitate long-term depression *in vivo*: role of tumour necrosis factor-α and the integrated stress response

**DOI:** 10.1093/braincomms/fcae333

**Published:** 2024-09-27

**Authors:** Neng-Wei Hu, Tomas Ondrejcak, Igor Klyubin, Yin Yang, Dominic M Walsh, Frederick J Livesey, Michael J Rowan

**Affiliations:** Department of Pharmacology & Therapeutics, School of Medicine, and Institute of Neuroscience, Trinity College, Dublin 2, Dublin, Ireland; Department of Physiology and Neurobiology, School of Basic Medical Sciences, Zhengzhou University, Zhengzhou 450001, China; Department of Pharmacology & Therapeutics, School of Medicine, and Institute of Neuroscience, Trinity College, Dublin 2, Dublin, Ireland; Department of Pharmacology & Therapeutics, School of Medicine, and Institute of Neuroscience, Trinity College, Dublin 2, Dublin, Ireland; Department of Pharmacology & Therapeutics, School of Medicine, and Institute of Neuroscience, Trinity College, Dublin 2, Dublin, Ireland; Department of Physiology and Neurobiology, School of Basic Medical Sciences, Zhengzhou University, Zhengzhou 450001, China; Laboratory for Neurodegenerative Research, Ann Romney Center for Neurologic Diseases, Brigham and Women’s Hospital and Harvard Medical School, Boston, MA 02115, USA; UCL Great Ormond Street Institute of Child Health, Zayed Centre for Research into Rare Disease in Children, University College London, London WC1N 1DZ, UK; Department of Pharmacology & Therapeutics, School of Medicine, and Institute of Neuroscience, Trinity College, Dublin 2, Dublin, Ireland

**Keywords:** Alzheimer’s disease, tau protein, amyloid-β, tumour necrosis factor-α, integrated stress response

## Abstract

Alzheimer’s disease is characterized by a progressive cognitive decline in older individuals accompanied by the deposition of two pathognomonic proteins amyloid-β and tau. It is well documented that synaptotoxic soluble amyloid-β aggregates facilitate synaptic long-term depression, a major form of synaptic weakening that correlates with cognitive status in Alzheimer’s disease. Whether synaptotoxic tau, which is also associated strongly with progressive cognitive decline in patients with Alzheimer’s disease and other tauopathies, also causes facilitation remains to be clarified. Young male adult and middle-aged rats were employed. Synaptotoxic tau and amyloid-β were obtained from different sources including (i) aqueous brain extracts from patients with Alzheimer’s disease and Pick’s disease tauopathy; (ii) the secretomes of induced pluripotent stem cell-derived neurons from individuals with trisomy of chromosome 21; and (iii) synthetic amyloid-β. *In vivo* electrophysiology was performed in urethane anaesthetized animals. Evoked field excitatory postsynaptic potentials were recorded from the stratum radiatum in the CA1 area of the hippocampus with electrical stimulation to the Schaffer collateral–commissural pathway. To study the enhancement of long-term depression, relatively weak low-frequency electrical stimulation was used to trigger peri-threshold long-term depression. Synaptotoxic forms of tau or amyloid-β were administered intracerebroventricularly. The ability of agents that inhibit the cytokine tumour necrosis factor-α or the integrated stress response to prevent the effects of amyloid-β or tau on long-term depression was assessed after local or systemic injection, respectively. We found that diffusible tau from Alzheimer’s disease or Pick’s disease patients’ brain aqueous extracts or the secretomes of trisomy of chromosome 21 induced pluripotent stem cell-derived neurons, like Alzheimer’s disease brain-derived amyloid-β and synthetic oligomeric amyloid-β, potently enhanced synaptic long-term depression in live rats. We further demonstrated that long-term depression facilitation by both tau and amyloid-β was age-dependent, being more potent in middle-aged compared with young animals. Finally, at the cellular level, we provide pharmacological evidence that tumour necrosis factor-α and the integrated stress response are downstream mediators of long-term depression facilitation by both synaptotoxic tau and amyloid-β. Overall, these findings reveal the promotion of an age-dependent synaptic weakening by both synaptotoxic tau and amyloid-β. Pharmacologically targeting shared mechanisms of tau and amyloid-β synaptotoxicity, such as tumour necrosis factor-α or the integrated stress response, provides an attractive strategy to treat early Alzheimer’s disease.

## Teaser

Hu *et al.* report that Alzheimer’s disease-related proteins tau and amyloid-β both facilitate synaptic long-term depression, a process by which synapses become less efficient in transmitting neuronal signals. Thus, targeting common pathways of both proteins could be a promising approach for Alzheimer’s disease treatment.

## Graphical Abstract

**Graphical abstract:**
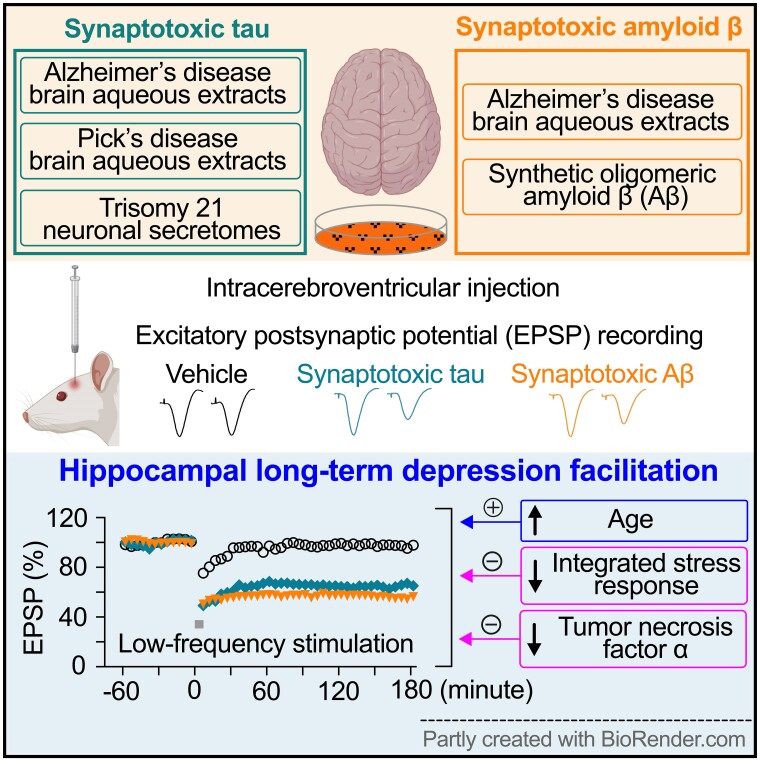

## Introduction

Many debilitating symptoms of Alzheimer’s disease are attributed to synaptic failure at both functional and structural levels.^1-4^ Studies of mechanisms underlying synaptopathy have evaluated the synaptotoxic potential of diffusible forms amyloid-β (Aβ) and, to a lesser extent, tau. Enduring changes in synaptic efficacy at glutamatergic synapses in vulnerable circuits, such as the entorhinal–hippocampal network, are very sensitive to disruption by Aβ. Exogenous application of diffusible forms of Aβ potently inhibits long-term potentiation (LTP)^5-7^ and enhances long-term depression (LTD)^8-11^ of synaptic transmission, supporting an Aβ-mediated synaptic depression model of early Alzheimer’s disease pathogenesis.^12-15^ In this model, the inhibition of LTP and promotion of LTD by Aβ, including those in aqueous Alzheimer’s disease brain extracts,^10,16^ trigger synaptic depression mechanisms that ultimately cause the gradual loss of synapses in early Alzheimer’s disease.

Tau is also strongly implicated in the synaptic defects in Alzheimer’s disease with positive correlations between the extent of tau pathology, cognitive impairment and glutamatergic synapse loss observed both post-mortem^17-21^ and recently *in vivo*.^22,23^ Interestingly, endogenous physiological tau has been reported to be required for certain forms of LTD^24-26^ (but see^27,28^) and Aβ-mediated inhibition of LTP^29^ (but see^30^), although how the synaptic depression model might be extended to include pathological tau remains to be clarified.^31-33^ Although tau is primarily an intracellular protein, we previously reported LTP impairing action by exogenous application of putative synaptotoxic tau from different sources including aqueous extracts of Alzheimer’s disease brain^34,35^ and Pick’s disease brain^36-38^ (mainly intracellular forms of tau) and secretomes of induced pluripotent stem cell (iPSC)-derived neurons from people with trisomy of chromosome 21 (Ts21)^39^ (mainly extracellular forms of tau). Given the diverse forms of tau, which have been shown to be synaptotoxic, we wondered if any of these species might affect LTD.

The onset of the prodromal stage of Alzheimer’s disease starts many decades earlier than the clinical diagnosis of dementia, which exponentially rises in people who are in their 80s.^40^ We therefore hypothesized that any shared synaptic depression-promoting action of Aβ and tau would be accentuated in middle-aged animals.^41^ Growing evidence indicates significant crosstalk between pro-inflammatory processes, in particular, the master pro-inflammatory cytokine tumour necrosis factor-α (TNF-α)^42^ and the integrated stress response (ISR) in Alzheimer’s disease.^43,44^ Given the potential importance of such pro-inflammatory processes and the ISR in synaptic disruption in Alzheimer’s disease,^45-47^ we investigated if targeting TNF-α or the ISR modulated any LTD-promoting actions of Aβ or tau.

## Materials and methods

### Animals and surgery

Young adult (2–3-month-old) and middle-aged (9–16-month-old) male Wistar and Lister Hooded rats were provided by the Comparative Medicine Unit of Trinity College Dublin. The animals were housed under a 12:12 h light–dark cycle (lights on at 8 a.m.) at room temperature (19–22°C) with access to food and water *ad libitum*. Prior to the acute experiments, animals were anaesthetized with urethane (1.5–1.6 g/kg, intraperitoneally, i.p.). Lignocaine (10 mg, 1% adrenaline, subcutaneously, s.c.) was injected over the area of the skull where electrodes and screws were to be implanted. The body temperature of the rats was maintained at 37–38°C with a feedback-controlled heating blanket. All efforts were made to minimize the number of animals used and their suffering. Totally 199 rats were used in this study. Animal care and experimental protocols followed the ARRIVE (Animal Research: Reporting of *In Vivo* Experiments) guidelines 2.0^48^ and were approved by the Health Products Regulatory Authority, Ireland, and the Animal Research Ethics Committee of Trinity College Dublin.

### Cannula implantation

Cannula implantation followed the protocols described in our previous studies.^10,39^ A stainless-steel cannula (22 gauge, 0.7 mm outer diameter) was located above the right lateral ventricle (1 mm lateral to the midline and 4 mm below the surface of the dura). For intracerebroventricular (i.c.v.) injection, an internal cannula (28 gauge, 0.36 mm outer diameter) was utilized, administering solutions of drugs or Aβ and tau at a rate of 1.5 μL/min. The placement of the cannula was verified post-mortem by observing the spread of ink dye following i.c.v. injection.

### *In vivo* electrophysiology

Electrodes were made and implanted as described previously.^10,39,49^ In brief, monopolar recording electrodes and twisted bipolar stimulating electrodes were crafted from Teflon-coated tungsten wires. Electrode sites were determined using stereotaxic coordinates relative to bregma: the recording electrode was positioned 3.4 mm posterior to bregma and 2.5 mm lateral to midline, and the stimulating electrode was located 4.2 mm posterior to bregma and 3.8 mm lateral to midline. Field excitatory postsynaptic potentials (EPSPs) were recorded from the stratum radiatum in the CA1 region of the right hippocampus, in response to stimulation of the ipsilateral Schaffer collateral–commissural pathway. Test EPSPs were elicited by a single square wave pulse (0.2 ms duration) at 0.033 Hz frequency, with an intensity that evoked a 50% maximum EPSP response. A peri-threshold low-frequency electrical stimulation (LFS) protocol, used to study the Aβ-mediated facilitation of LTD,^10^ consisted of 300 pulses (0.2 ms duration) at 1 Hz, with an intensity that evoked 95% maximum amplitude. Throughout the experiments, none of the conditioning stimulation protocols induced any detectible abnormal changes in background EEG, which was continuously monitored from the hippocampus.

### Compounds and antibodies

Etanercept (Enbrel), a soluble dimeric form of the TNF receptor that very selectively binds and neutralizes TNF,^50^ was purchased from the Pharmacy Department, St. James’s Hospital, Dublin, Ireland. Etanercept was pre-injected (i.c.v.) immediately before the injection of Aβ or tau. Tau5 (Cat. No. 80601; binding epitope at amino acids 210–241 of full-length tau) and isotype control 6E10 (Cat. No. 803015; mouse IgG1, anti-Aβ N-terminus) antibodies were purchased from BioLegend. *Trans*-*N*,*N*′-(cyclohexane-1,4-diyl)bis(2-(4-chlorophenoxy) acetamide (ISRIB, Sigma, SML0843), a very potent and highly selective drug-like small molecule ISR inhibitor that acts by reversing the effects of eukaryotic initiation factor 2α (eIF2α) phosphorylation,^51^ was dissolved in dimethyl sulfoxide (DMSO) with gentle warming in a 40°C water bath and vortexed until the solution became clear. Then the solution was diluted in polyethylene glycol 400 (PEG400) and again gently warmed in a 40°C water bath and vortexed. The solution was prepared freshly and diluted in warm saline (37°C) before injection. A 1:1 mixture of DMSO and PEG400 in saline was used as vehicle control. The dose and timing choices for injection of the different agents were based on previous reports. For co-injection of antibodies, the brain extracts were co-incubated for 5 min prior to i.c.v. injection using doses based on prior findings.^34,35^ The dose of etanercept (50 µg in 5 µL, i.c.v.) used here was previously reported to restore disrupted LTP in freely moving transgenic rats overexpressing Alzheimer’s disease-associated β-amyloid precursor protein at a pre-plaque stage of amyloidosis.^52^ Similarly, the ISRIB dosing was based on our previous study of its pharmacokinetics in live rats^53^ and the report that 1 day after a single ISRIB treatment systemically (2.5 mg/kg, i.p.), age-related changes in CA1 pyramidal neuron function and structure improved.^54^

### Aβ- and tau-containing aqueous extracts of the human brain

Human brain tissue was used in accordance with the guidelines of Trinity College Dublin Faculty of Health Science Ethics Committee (under approval 16014) and the Harvard University Partner’s Institutional Review Board (Protocol: Walsh BWH 2011). The detailed methods of preparation of Alzheimer’s disease brain aqueous extracts were described previously.^34,35^ Whereas the vast majority of studied extracts inhibited LTP in an Aβ-dependent manner, recently we found that ∼1 in 15 Alzheimer’s disease brain extracts retained their deleterious effects on LTP after depletion of Aβ. These extracts inhibited LTP in a tau-dependent manner, an effect abrogated by selective depletion of tau.^34^ In this study, we used frozen brain tissue obtained from four cases, three of whom died of end-stage Alzheimer’s disease and one with Pick’s disease (PiD1) (Table 1). Two Alzheimer’s disease brain extracts, referred to as ‘AD1’ and ‘AD2’ used in our previous report,^34^ inhibit LTP in a tau-dependent manner. The third Alzheimer’s disease brain extract, referred to as ‘AD7’ used in a separate report,^35^ inhibits LTP in an Aβ-dependent manner (Table 1). Tissue from ‘AD1’ was obtained from the Massachusetts ADRC Neuropathology Core, Massachusetts General Hospital, and tissue from ‘AD2’ and ‘AD7’ was acquired from Tissue Solutions, UK. ‘AD1’ was a 75-year-old woman, ‘AD2’ was a 79-year-old male, ‘AD7’ was a 95-year-old woman, and ‘PiD1’ was a 79-year-old male. All cases met post-mortem and clinical diagnostic criteria for the relevant disorder.

**Table 1:** Previously reported effectiveness of Aβ ID with AW7 and tau ID with Tau5 on LTP inhibition by the brain extracts and Ts21 secretomes used in the present studies

Figure in this study	Patient’s code	Immunodepletion information	Percent reduction of Aβ or tau	LTP inhibition	Reference
Fig. 1	AD2	Mock ID (PIS)		Yes	Ondrejcak *et al.* (2018)^34^
		Aβ ID (AW7)	nd	Yes	Ondrejcak *et al.* (2018)^34^
Figs. 2, 6, 7	AD1	Aβ ID (AW7)	∼82%	Yes	Ondrejcak *et al.* (2018)^34^
		Mock ID (46-4)		Yes	Ondrejcak *et al.* (2018)^34^
		Tau ID (Tau5)	∼82%	No	Ondrejcak *et al.* (2018)^34^
Fig. 3	PiD1	Mock ID (46-4)		Yes	Ondrejcak *et al.* (2023)^35^
		Tau ID (Tau5)	∼48%	No	Ondrejcak *et al.* (2023)^35^
Fig. 4	Ts21	Aβ ID (AW7)	<LLoQ	Yes	Hu *et al.* (2018)^39^
		Mock ID (46-4)		Yes	Hu *et al.* (2018)^39^
		Tau ID (Tau5)	∼79%	No	Hu *et al.* (2018)^39^
Figs. 5–7	AD7	Mock ID (PIS)		Yes	Ondrejcak *et al.* (2023)^35^
		Aβ ID (AW7)	∼98%	No	Ondrejcak *et al.* (2023)^35^

nd, not determined; LLoQ, the lower limit of quantitation; PIS, pre-immune serum.

### Synaptotoxic tau-containing secretomes from Ts21 iPSC-derived neurons

The Ts21 iPSC line was generated from a fibroblast biopsy of an individual with Down syndrome and used in accordance with the UK Code of Practice for the Use of Human Stem Cell Lines. The detailed methods of iPSC and cortical neuron culture generation, secretome collection and processing were described previously.^39,55^ Briefly, secretomes were collected at 48 h intervals between Days 70 and 80. Secretomes were clarified by three-step centrifugation (200 × *g* for 10 min, 10 000 × g for 30 min and 100 000 × g for 2 h), intended to remove dead cells, debris and exosomes, and dialyzed against artificial cerebrospinal fluid (aCSF), to remove small molecules, before freezing at −80°C. Secretomes were characterized by analytical size exclusion chromatography (SEC) and western blot analysis. Detailed information was presented in our previous report (Supplemental Experimental Procedures).^39^

### Patient-derived sample immunodepletion of Aβ or tau

The detailed methods of Aβ or tau immunodepletion (ID) of brain aqueous extracts and secretomes from Ts21 iPSC-derived neurons were described previously.^34,35^

For Aβ immunodepletion, one portion of Alzheimer’s disease (AD1, AD2 and AD7) brain extract was immunodepleted by three rounds of 12 h incubations with the anti-Aβ polyclonal antibody, AW7 and Protein A Sepharose beads at 4°C. A second portion of Alzheimer’s disease brain extract was treated identically but incubated with pre-immune rabbit serum (PIS) plus Protein A Sepharose beads to yield ‘mock ID’ samples. Aβ immunodepletion of Ts21 secretomes was performed by two rounds of 12 h incubation with AW7 and Protein A Sepharose beads at 4°C. Pre-immune serum was used as an identical control. Samples were cleared of beads, and aliquots were stored at −80°C.

For tau immunodepletion, one portion of AD1 brain extract was immunodepleted by two rounds of 16 h incubations with the anti-tau mAb Tau5 (10 µg) and Protein G Agarose (10 µL) beads at 4°C. A second portion of AD1 extract was treated identically with the control mAb 46-4 and Protein G Agarose beads at 4°C. Extract of PiD1 (0.5 ml aliquots) was immunodepleted of tau by three rounds (8, 16 and 8 h) of incubation with Tau5 (10 µg) and Protein G Agarose (10 µL) beads at 4°C. Mock ID was performed identically with isotype control mAb 46-4 (10 µg) and Protein G Agarose (10 µL) beads at 4°C. Tau immunodepletion of Ts21 secretomes was performed by two rounds of 12 h incubation with Tau5 (10 µg) and Protein G Agarose beads at 4°C. The isotype control mAb 46-4 (10 µg) and Protein G Agarose beads were used as a control in tau immunodepletion. The Tau5- and 46-4-treated samples were cleared of beads and then incubated with Protein G Agarose alone to remove residual IgG. The aliquots were stored at −80°C before using (Table 1). The volumes of brain extract or secretome to be injected were based on our previously published findings^34,35,39^ and determined in dose-ranging pilot experiments. Furthermore, tau characterization and quantification were presented in our previous reports using these brain extracts^35^ and secretomes.^39^

### Preparation of oligomeric Aβ

Aβ_1–42_ (human sequence) was synthesized and purified using reverse-phase HPLC by Dr. James I. Elliott at the ERI Amyloid laboratory, Oxford, CT, USA. A single batch of oligomeric Aβ was prepared with the same protocol as described previously.^10^ Briefly, the peptide was dissolved in ice-cold 1,1,1,3,3,3-hexafluoroisopropanol (HFIP) to a concentration of 1 mM, sonicated for 10 min and left to stand at room temperature for 1 h. The HFIP was evaporated using a stream of dry air/N_2_ to produce a clear, homogenous peptide film. This film was dissolved in anhydrous DMSO to produce a 5 mM solution and then diluted to 100 μM in phenol red-free Ham’s F12 media and vortexed for 15 s. After 18 h incubation at 4°C, the sample was centrifuged at 16 000 × *g* for 5 min to remove large preformed aggregates, and the concentration of oligomeric Aβ in the supernatant was determined using NanoDrop with the extinction coefficient *ε*_275_ = 1361 M^−1^cm^−1^.^56^ Oligomeric Aβ in the sample was characterized by analytical SEC (TSKgel superSW2000). Aliquots of Aβ solution were immediately frozen on dry ice and stored at −80°C.

### Data analysis

**Figure 1:**
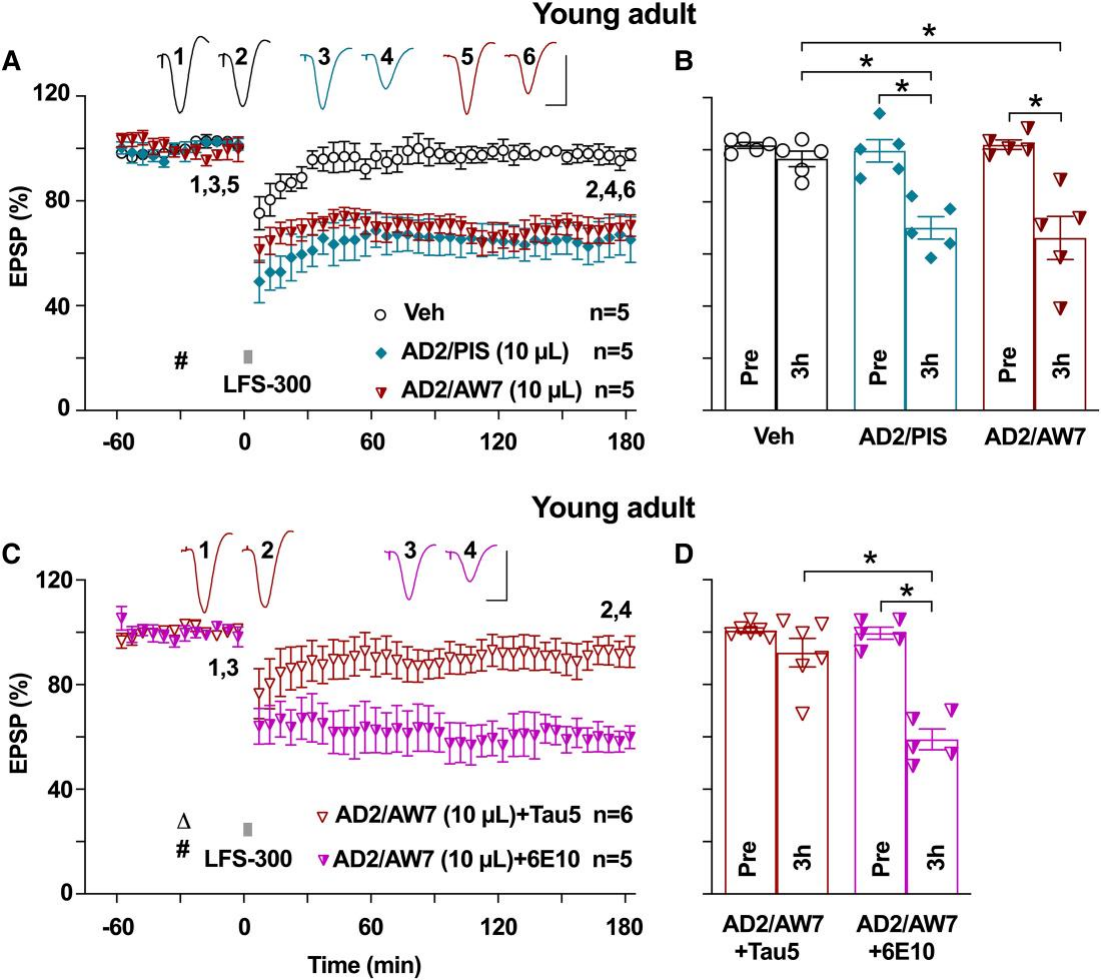
**Synaptotoxic tau in Alzheimer’s disease brain aqueous extracts facilitates hippocampal LTD in young rats.** (**A**) The application of weak LFS (bar, LFS-300, 300 pulses at 1 Hz) did not induce stable LTD in young adult (2–3-month-old) anaesthetized rats. Acute i.c.v. injection of an Alzheimer’s disease brain aqueous extract containing LTP-disrupting tau,^34^ mock-immunodepleted with pre-immune serum (AD2/PIS, 10 µL, i.c.v., hash), enabled the induction of a robust and stable LTD by the weak LFS-300 protocol. After immunodepleting Aβ with a polyclonal antibody, this extract (AD2/AW7, 10 µL, i.c.v., hash) still facilitated LTD by LFS-300. (**B**) At 3 h post-LFS, the EPSP measured 96.5 ± 3.0% in the vehicle control group (*n* = 5, *P* > 0.05 compared with Pre; paired *t*), 66.2 ± 8.3% in the AD2 mock-immunodepleted group (AD2/PIS, *n* = 5, *P* < 0.05 compared with Pre and *P* < 0.05 compared with the vehicle group; paired *t* and one-way ANOVA-Bonferroni) and 70.0 ± 4.3% in the Aβ-ID group (AD2/AW7, *n* = 5, *P* < 0.05 compared with Pre and *P* < 0.05 compared with the vehicle group; paired *t* and one-way ANOVA-Bonferroni). (**C**) Co-injection of this tau-containing Alzheimer’s disease brain aqueous extract with the anti-tau antibody Tau5 prevented the facilitation of LTD by LFS-300. In contrast, co-injection of the same extract with the anti-Aβ antibody 6E10 enabled the induction of LTD by LFS-300. Open triangle, Tau5 or 6E10 antibodies; hash, ‘AD2’ brain extract. (**D**) At 3 h post-LFS, the EPSP measured 92.2 ± 5.5% in the Tau5 co-injected group (AD2/AW7 + Tau5, *n* = 6, *P* > 0.05 compared with Pre; paired *t*) and 59.1 ± 4.0% in the 6E10 co-injected group (AD2/AW7 + 6E10, *n* = 5, *P* < 0.05 compared with Pre, paired *t*; *P* < 0.05 compared with AD2/AW7 + Tau5, two-way ANOVA-Bonferroni). Calibration bars for EPSP traces: vertical, 2 mV; horizontal, 10 ms. Each data point represents the average of 10 EPSP sweeps (5 min), and the magnitude of LTD is expressed as the percentage of pre-LFS baseline EPSP amplitude in **A** and **C**.

**Figure 2:**
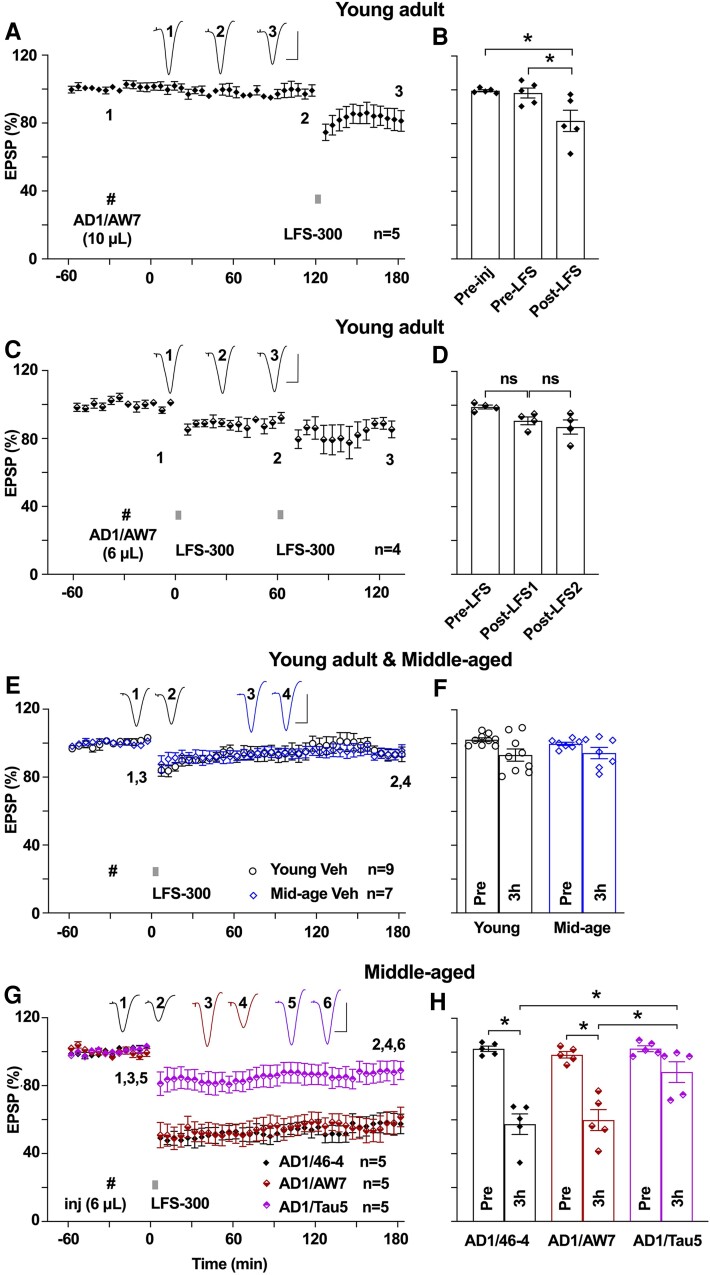
**Alzheimer’s disease brain tau facilitates hippocampal LTD in an age-dependent manner.** (**A**) In young rats, i.c.v. injection of another Alzheimer’s disease brain aqueous extract that contains LTP-disrupting tau^34^ (AD1/AW7, 10 µL, i.c.v., hash) did not affect baseline synaptic transmission for 2.5 h and enabled the induction of a small but significant LTD by the weak LFS-300 protocol. This extract had been immunodepleted of Aβ with a polyclonal antibody. As summarized in **B**, the EPSP measured 98.1 ± 2.9% 2.5 h after i.c.v. injection of AD1/AW7 (*n* = 5, *P* > 0.05 compared with Pre-injection, RM one-way ANOVA-Bonferroni) and 81.7 ± 6.3% 1 h post-LFS-300 (*P* < 0.05 compared with Pre-injection and Pre-LFS, RM one-way ANOVA-Bonferroni). (**C**) However, a lower dose (6 µL) of the same extract of Alzheimer’s disease brain did not enable the induction of LTD even after application of LFS-300 twice in young rats. As summarized in **D**, the EPSP measured 90.8 ± 2.3% at 1 h post-LFS1 (*n* = 4, *P* > 0.05 compared with Pre, RM one-way ANOVA-Bonferroni) and 87.2 ± 4.1% at 1 h post-LFS2 (*P* > 0.05 compared with pre- and post-LFS1, RM one-way ANOVA-Bonferroni). (**E**) Similar to young animals, the application of LFS-300 did not induce stable LTD in middle-aged (9–16-month-old) anaesthetized rats. (**F**) At 3 h post-LFS, the EPSP measured 93.3 ± 3.6% in young rats (young Veh, *n* = 9, *P* > 0.05 compared with Pre; paired *t*), 94.5 ± 3.3% in the middle-aged group (mid-age Veh, *n* = 7, *P* > 0.05 compared with Pre, paired *t*; *P* > 0.05 compared with young animals, two-way ANOVA-Bonferroni). (**G**) In middle-aged rats, the lower dose (6 µL) of the same Alzheimer’s disease brain extract that had been immunodepleted of Aβ (AD1/AW7) enabled the induction of robust and persistent LTD by LFS-300. In contrast, the same extract of Alzheimer’s disease brain that had been immunodepleted of tau, using the anti-tau antibody Tau5 (AD1/Tau5), failed to enable the induction of LTD by LFS-300. In contrast, samples mock-immunodepleted with the isotype control mAb 46-4 (AD1/46-4) facilitated LTD. (**H**) As summarized at 3 h, the EPSP measured 57.4 ± 6.1% in the AD1/46-4 group (*n* = 5, *P* < 0.05 compared with Pre, paired *t*), 59.9 ± 6.2% in the AD1/AW7 group (*n* = 5, *P* < 0.05 compared with Pre, and *P* > 0.05 compared with the AD1/46-4 group; paired *t* and one-way ANOVA-Bonferroni) and 88.3 ± 6.2% in the AD1/Tau5 group (*n* = 5, *P* > 0.05 compared with Pre, *P* < 0.05 compared with the AD1/46-4 group and AD1/AW7 group; paired *t* and one-way ANOVA-Bonferroni). Calibration bars for EPSP traces: vertical, 2 mV; horizontal, 10 ms. Each data point represents the average of 10 EPSP sweeps (5 min), and the magnitude of LTD is expressed as the percentage of pre-LFS baseline EPSP amplitude in **A**, **C**, **E** and **G**.

**Figure 3:**
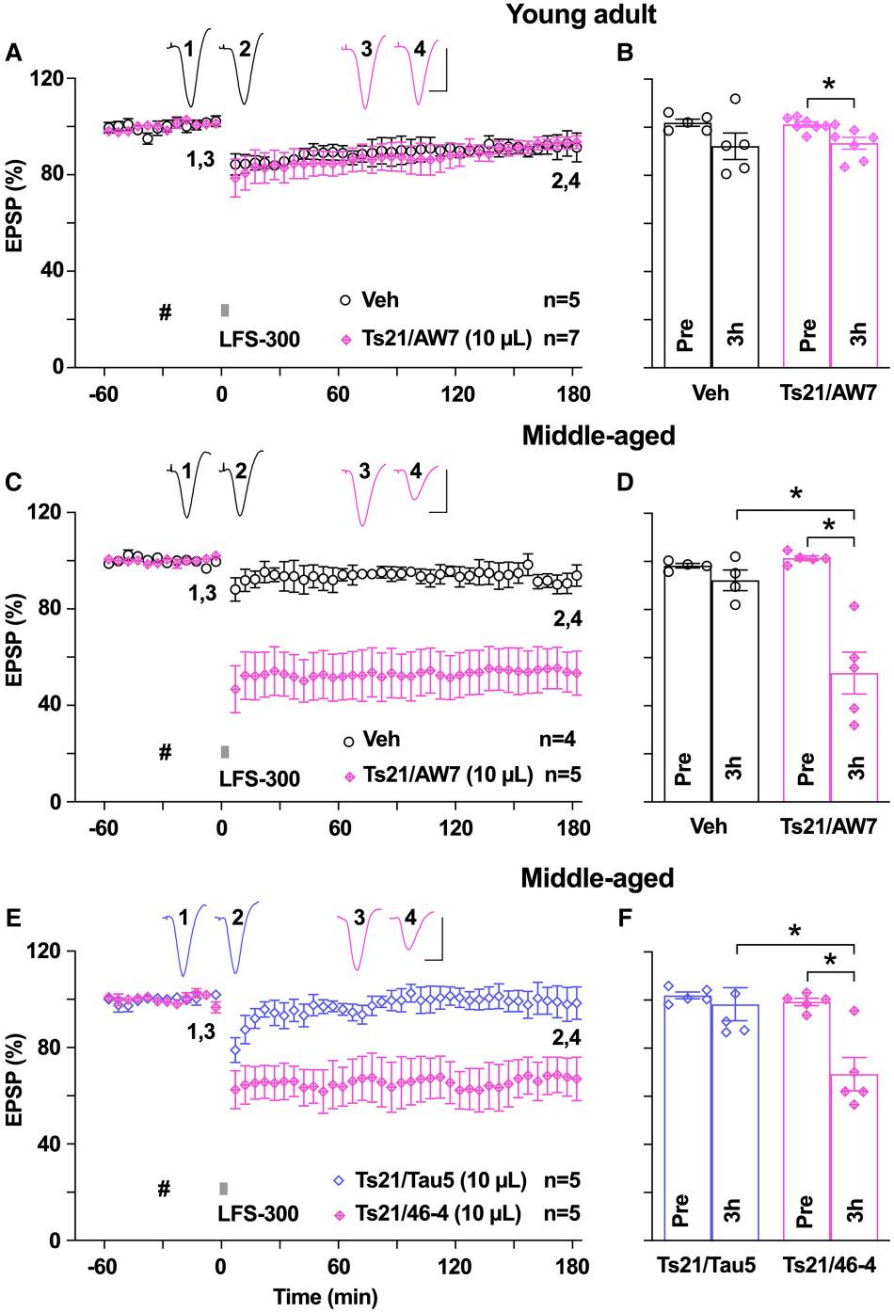
**Extracellular soluble tau in Ts21 secretomes facilitates hippocampal LTD in an age-dependent manner.** (**A**) The application of LFS-300 did not induce obvious LTD in young rats after i.c.v. injection of either vehicle (PBS) or secretome of Ts21 iPSC-derived cortical neurons (Ts21/AW7, 10 µL, hash). The secretome had been immunodepleted of Aβ with the polyclonal antibody AW7. As summarized in **B**, the EPSP measured 92.2 ± 5.5% in the vehicle control group (*n* = 5, *P* > 0.05 compared with Pre; paired *t*) and 93.4 ± 2.5% in the Ts21/AW7 group (*n* = 7, *P* > 0.05 compared with Pre, and *P* > 0.05 compared with the vehicle control group; paired *t* and two-way ANOVA-Bonferroni) 3 h post-LFS. (**C**) In contrast, the same volume of this secretome (Ts21/AW7) enabled the induction of robust and persistent LTD by LFS-300 in middle-aged rats. As summarized in **D**, at 3 h post-LFS, the EPSP measured 92.2 ± 4.3% in the vehicle control group (*n* = 4, *P* > 0.05 compared with Pre; paired *t*) and 53.6 ± 8.7% in the Ts21/AW7 group (*n* = 5, *P* < 0.05 compared with Pre, and *P* < 0.05 compared with the vehicle control group; paired *t* and two-way ANOVA-Bonferroni). (**E**) Immunodepletion of tau with the anti-tau antibody Tau5 (Ts21/Tau5) prevented the facilitation of LTD by Ts21 secretome in middle-aged rats whereas secretome that had been mock-immunodepleted with a control antibody 46-4 (Ts21/46-4) enabled the induction of robust and persistent LTD by LFS-300. (**F**) As summarized at 3 h, the EPSP measured 69.2 ± 6.9% in the Ts21/46-4 group (*n* = 5, *P* < 0.05 compared with Pre, paired *t*) and 98.2 ± 6.87% in the Ts21/Tau5 group (*n* = 5, *P* > 0.05 compared with Pre, and *P* < 0.05 compared with the Ts21/46-4 group; paired *t* and two-way ANOVA-Bonferroni). Calibration bars for EPSP traces: vertical, 2 mV; horizontal, 10 ms. Each data point represents the average of 10 EPSP sweeps (5 min), and the magnitude of LTD is expressed as the percentage of pre-LFS baseline EPSP amplitude in **A**, **C** and **E**.

**Figure 4:**
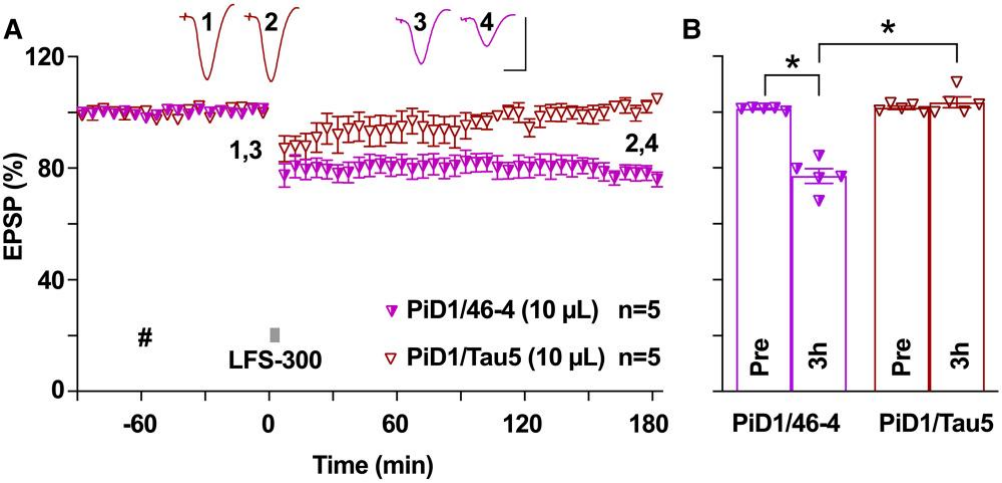
**Synaptotoxic tau in Pick’s disease brain aqueous extracts facilitates hippocampal LTD in young rats.** (**A**) In young animals, acute i.c.v. injection of a Pick’s disease brain aqueous extract containing LTP-disrupting tau,^35^ mock-immunodepleted with the isotype control mAb 46-4 (PiD1/46-4, 10 µL, i.c.v., hash), enabled the induction of a robust and stable LTD by the weak LFS-300, whereas immunodepletion of tau with the anti-tau antibody Tau5 (PiD1/Tau5, 10 µL, i.c.v., hash) prevented the facilitation of LTD. Each data point represents the average of 10 EPSP sweeps (5 min), and the magnitude of LTD is expressed as the percentage of pre-LFS baseline EPSP amplitude. (**B**) At 3 h post-LFS, the EPSP measured 77.1 ± 2.6% in the PiD1/46-4 group (*n* = 5, *P* < 0.05 compared with Pre, paired *t*) and 103.5 ± 2.0% in the PiD1/Tau5 group (*n* = 5, *P* > 0.05 compared with Pre, paired *t*; *P* < 0.05 compared between two groups, two-way ANOVA-Bonferroni). Calibration bars for EPSP traces: vertical, 2 mV; horizontal, 10 ms.

**Figure 5:**
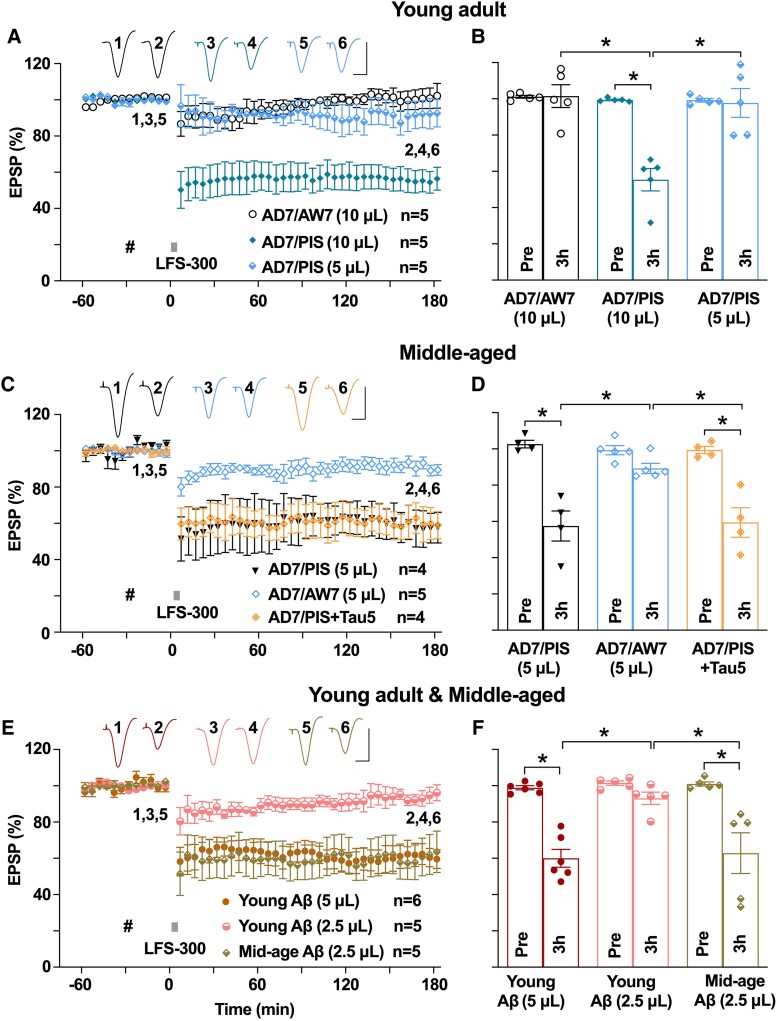
**Alzheimer’s disease brain synaptotoxic Aβ and synthetic Aβ facilitates hippocampal LTD in an age-dependent manner.** (**A**) Acute i.c.v. injection of an Alzheimer’s disease brain aqueous extract containing LTP-disrupting Aβ^34^ (AD7/PIS, 10 µL) enabled the induction of robust and stable LTD by the weak LFS-300 protocol in young animals, while i.c.v. injection of Aβ-ID (AD7/AW7, 10 µL) extract failed to facilitate LTD. However, half this volume of the same brain extract (AD7/PIS, 5 µL) did not enable the induction of LTD by LFS-300. (**B**) As summarized at 3 h, the EPSP measured 101.5 ± 6.3% in the AD7/AW7 (10 µL) group (*n* = 5, *P* > 0.05 compared with Pre; paired *t*), 55.4 ± 6.2% in the AD7/PIS (10 µL) group (*n* = 5, *P* < 0.05 compared with Pre and the AD7/AW7 group; paired *t* and one-way ANOVA-Bonferroni) and 97.8 ± 8.0% in the AD7/PIS (5 µL) group (*n* = 5, *P* > 0.05 compared with Pre and the AD7/AW7 group; *P* < 0.05 compared with 10 µL-treated animals, paired *t* and one-way ANOVA-Bonferroni). (**C**) In middle-aged rats, the lower volume (5 µL) of the same Alzheimer’s disease brain extract (AD7/PIS) enabled the induction of LTD by LFS-300 and immunodepletion of Aβ using a polyclonal anti-Aβ antibody (AD7/AW7) prevented the facilitation of LTD. In contrast, co-injection of the anti-tau antibody Tau5 (AD7/PIS + Tau5) failed to prevent the facilitation of LTD. (**D**) As summarized at 3 h, the EPSP measured 49.9 ± 10.0% in the AD7/PIS (5 µL)-injected middle-aged rats (*n* = 5, *P* < 0.05 compared with Pre; paired *t*), 89.3 ± 2.8% in the AD7/AW7 (5 µL) group (*n* = 5, *P* > 0.05 compared with Pre, and *P* < 0.05 compared with the AD7/PIS group; paired *t* and one-way ANOVA-Bonferroni) and 59.5 ± 8.1% in the AD7/PIS + Tau5 group (*n* = 5, *P* < 0.05 compared with Pre, and *P* > 0.05 compared with the AD7/PIS group; paired *t* and one-way ANOVA-Bonferroni). (**E**) In young rats, whereas acute i.c.v. injection of 585 pmol (5 µL) synthetic Aβ facilitated the induction of a robust and persistent LTD by LFS-300, injection of half this dose (292.5 pmol in 2.5 µL) failed to facilitate LTD. In middle-aged rats, in contrast, the lower dose of Aβ enabled the induction of robust LTD by the same weak conditioning stimulation protocol. (**F**) As summarized at 3 h, the EPSP measured 60.0 ± 4.9% in higher dose (585 pmol in 5 µL) Aβ-injected young rats (*n* = 6, *P* < 0.05 compared with Pre; paired *t*), 93.0 ± 3.4% in lower dose (292.5 pmol in 2.5 µL) Aβ-injected young rats (*n* = 5, *P* > 0.05 compared with Pre and *P* < 0.05 compared with the young (5 µL) group; paired *t* and one-way ANOVA-Bonferroni) and 62.8 ± 11.2% in lower dose (292.5 pmol in 2.5 µL) Aβ-injected middle-aged rats (*n* = 5, *P* < 0.05 compared with Pre and *P* < 0.05 compared with lower dose Aβ-injected young rats; paired *t* and one-way ANOVA-Bonferroni). Calibration bars for EPSP traces: vertical, 2 mV; horizontal, 10 ms. Each data point represents the average of 10 EPSP sweeps (5 min), and the magnitude of LTD is expressed as the percentage of pre-LFS baseline EPSP amplitude in **A**, **C** and **E**.

**Figure 6:**
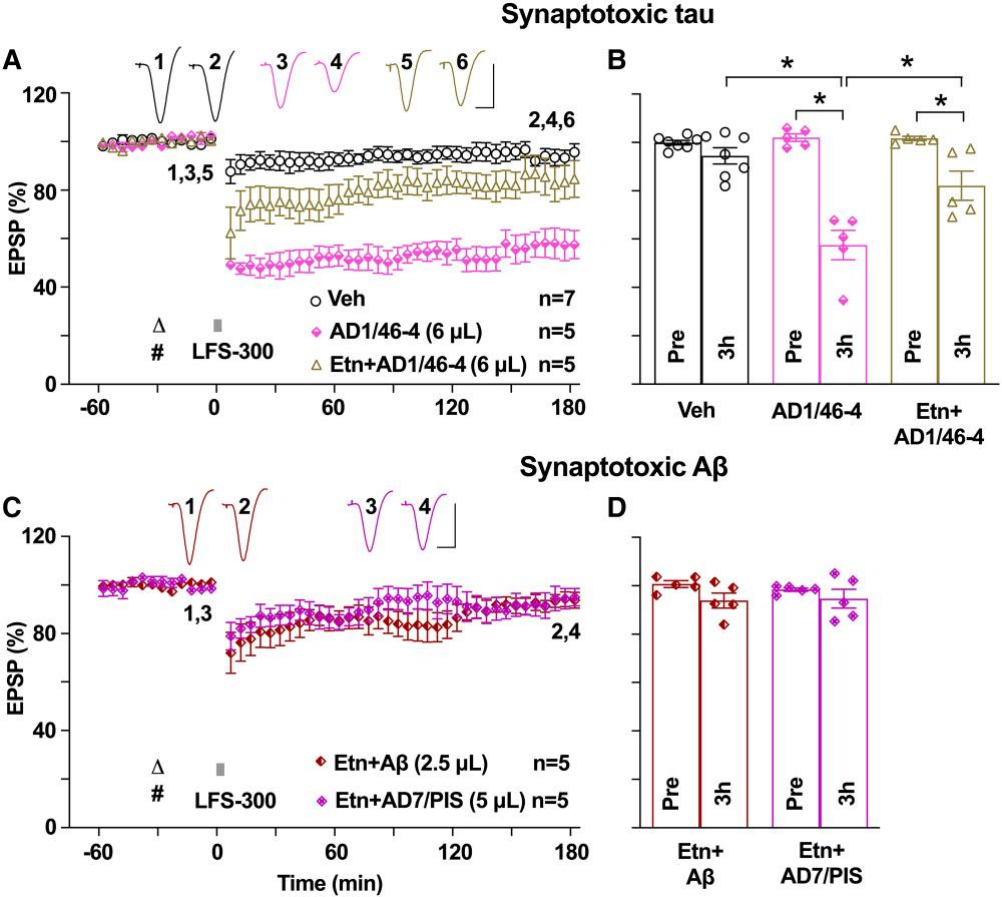
**The TNF-α inhibitor etanercept ameliorates the facilitation of LTD by synaptotoxic tau and Aβ in middle-aged rats.** (**A**) Pre-injection of etanercept (Etn, 50 µg/5 µL, i.c.v.) reduced the facilitation of LTD by i.c.v. injection of synaptotoxic tau-containing Alzheimer’s disease brain extract AD1/46-4 (6 µL) in middle-aged rats. Triangle, etanercept; hash, AD1/46-4. (**B**) As summarized at 3 h, the EPSP measured 82.2 ± 6.0% in the Etn + AD1/46-4 group [*n* = 5, *P* > 0.05 compared with the vehicle control group (94.5 ± 3.3%, *n* = 7) and *P* < 0.05 compared with the AD1/46-4 group (57.4 ± 6.1%, *n* = 5), one-way ANOVA-Bonferroni]. (**C**) The same dose of etanercept prevented LTD facilitation by both synthetic Aβ (292.5 pmol in 2.5 µL) and synaptotoxic Aβ-containing Alzheimer’s disease brain extract (AD7/PIS, 5 µL) in middle-aged rats. (**D**) As summarized at 3 h, the EPSP measured 93.9 ± 3.1% in the Etn + Aβ group (*n* = 5, *P* > 0.05 compared with Pre, paired *t*) and 94.7 ± 3.9% in the Etn + AD7/PIS group (*n* = 5, *P* > 0.05 compared with Pre, paired *t*; *P* > 0.05 compared between two groups, two-way ANOVA-Bonferroni). Calibration bars for EPSP traces: vertical, 2 mV; horizontal, 10 ms. Each data point represents the average of 10 EPSP sweeps (5 min), and the magnitude of LTD is expressed as the percentage of pre-LFS baseline EPSP amplitude in **A** and **C**.

**Figure 7:**
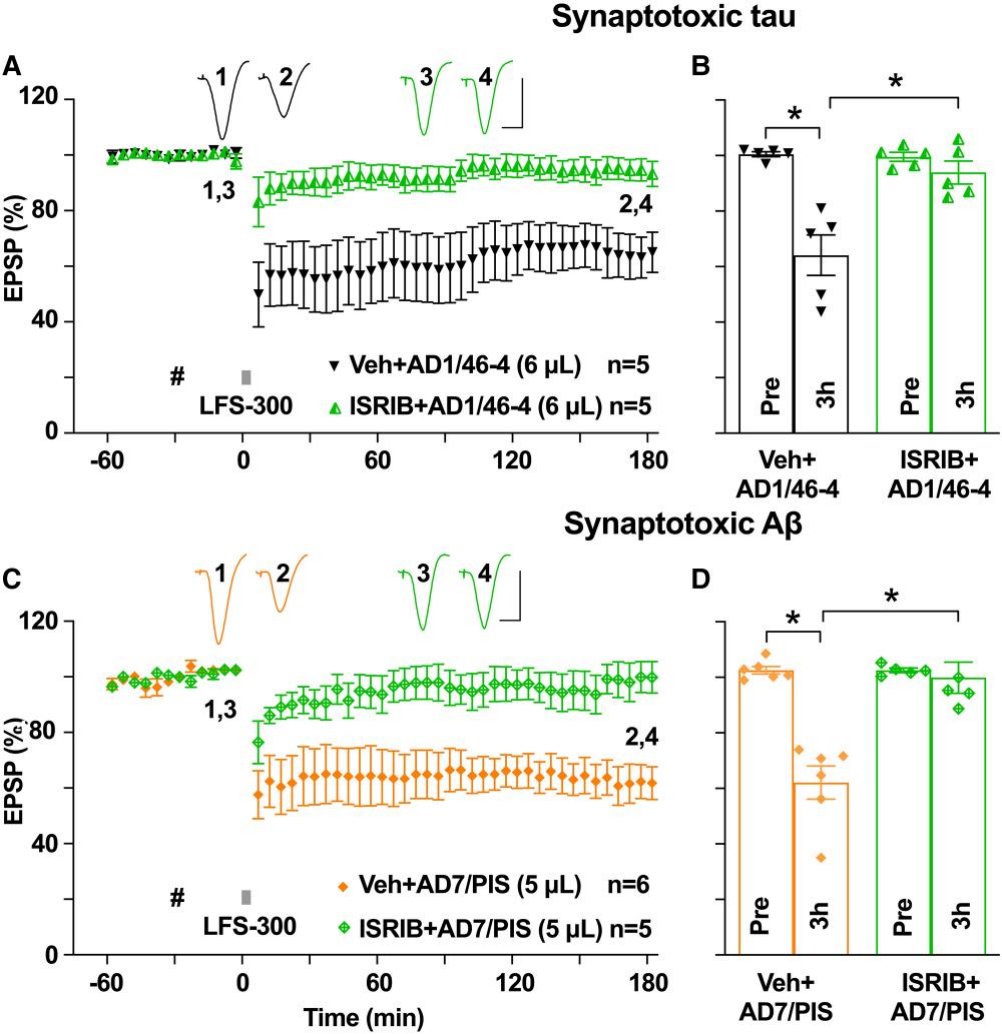
**ISRIB prevents the facilitation of LTD by Alzheimer’s disease brain tau and Aβ in middle-aged rats.** (**A**) Systemic pre-treatment of ISRIB (24 h Pre, 2.5 mg/kg, i.p.) completely abolished the facilitation of LTD by AD1/46-4 (6 µL) in middle-aged rats. (**B**) Summary at 3 h (95.1 ± 7.1% in the ISRIB-treated group, *n* = 4, *P* > 0.05 compared with Pre and *P* < 0.05 compared with the Veh + AD1/46-4 group; paired *t* and two-way ANOVA-Bonferroni). (**C**) Acute i.c.v. injection of the lower volume of diffusible Aβ-containing Alzheimer’s disease brain extract (AD7/PIS, 5 µL) enabled LTD by LFS-300. Systemic pre-treatment of ISRIB (24 h Pre, 2.5 mg/kg, i.p.) abolished LTD facilitation by AD7/PIS in middle-aged rats. (**D**) Summary at 3 h (99.9 ± 5.6% in the ISRIB-treated group, *n* = 5, *P* > 0.05 compared with Pre and *P* < 0.05 compared with the Veh + AD7/PIS group; paired *t* and two-way ANOVA-Bonferroni). Hash, AD1/46-4 or AD7/PIS. Calibration bars for EPSP traces: vertical, 2 mV; horizontal, 10 ms. Each data point represents the average of 10 EPSP sweeps (5 min), and the magnitude of LTD is expressed as the percentage of pre-LFS baseline EPSP amplitude in **A** and **C**.

The magnitude of LTD is expressed as the percentage of pre-LFS baseline EPSP amplitude (± SEM). The EPSP data are grouped into 5 min epochs (average of 10 sweeps) for graphing purposes. The last 10 min prior to LFS was used to calculate the ‘Pre’-induction EPSP amplitude. Unless otherwise stated, the magnitude of LTD was measured over the last 10 min at the end of recording ‘3 h’ after LFS. Control experiments were interleaved randomly throughout, and no data were excluded. To compare between two groups, repeated measures two-way ANOVA with Bonferroni *post hoc* test was used (Figs. 1D, 2F, 3B,D,F, 4B, 6D, 7B and D). To compare between groups of three or more, one-way ANOVA with Bonferroni multiple comparisons was used (Figs. 1B, 2H, 5B,D,F, and 6B). A two-tailed paired Student’s *t*-test (paired *t*) was used to compare between ‘Pre’ and ‘3 h’ within groups (Figs. 1B,D, 2F,H, 3B,D,F, 4B, 5B,D,F, 6B,D, 7B and D). Repeated measures (RM) one-way ANOVA was used to compare three or more treatments in one group (Fig. 2B and D). Some experiments on the effect of Ts21 secretomes on LTD were performed blindly. A *P* < 0.05 was considered as statistically significant. All data were evaluated and graphed using Prism 9.0 (GraphPad Inc., San Diego, CA, USA).

## Results

### Alzheimer’s disease brain-derived synaptotoxic tau facilitates LTD in an age-dependent manner

Because certain forms of tau are potent synaptotoxins,^17,19-23^ we wondered if tau present in Alzheimer’s disease brain aqueous extracts would, similar to Aβ, facilitate LTD. Previously, we reported that a small number (∼1 in 15) of Alzheimer’s disease brain aqueous extracts disrupt LTP in a tau-dependent and Aβ-independent manner.^34^ Before testing the facilitation of LTD, we first confirmed that a relatively weak LFS conditioning protocol^10^ was just below the threshold for inducing stable hippocampal LTD *in vivo*. Thus, the application of 300 high-intensity pulses at 1 Hz (LFS-300) failed to induce stable LTD at CA3-to-CA1 synapses in young adult (2–3 months of age) anaesthetized rats (Fig. 1A and B). Next, we tested the effect of one of these synaptotoxic tau-containing extracts (‘AD2’) on LTD in young adult rats (Table 1). We found that the weak LFS protocol induced robust and stable LTD after i.c.v. injection (10 µL) of the extract that had been mock-immunodepleted (with pre-immune serum) (Fig. 1A and B). Moreover, the same volume of this ‘AD2’ extract previously immunodepleted with anti-Aβ polyclonal antibody, AW7, still facilitated a robust and stable LTD (Fig. 1A and B). In contrast, co-injection of a mAb targeting the mid-region of tau, Tau5, prevented the LTD facilitation caused by ‘AD2’. Importantly, co-injection of the isotype-matched anti-Aβ antibody 6E10 did not (Fig. 1C and D). To further assess the role of tau in mediating the facilitation of LTD by extracts of Alzheimer’s disease brain, which inhibit LTP in a tau-dependent manner, we tested another, similar, extract (‘AD1’)^34^ (Table 1). After i.c.v. injection of 10 µL of AD1 that had been immunodepleted of Aβ, baseline synaptic transmission remained stable for 2.5 h, and the application of LFS-300 induced significant LTD (Fig. 2A and B), consistent with the proposal that tau in certain Alzheimer’s disease brain extracts can facilitate LTD.

Because ageing-related processes are strongly implicated in promoting cognitive decline in Alzheimer’s disease and other tauopathies, we wondered if the facilitation of LTD by synaptotoxic tau-containing Alzheimer’s disease brain extracts was age-dependent. Having seen the facilitation of LTD by acute i.c.v. injection of 10 µL ‘AD1’ sample, we reduced the volume of the injection to 6 µL, which failed to enable LTD by LFS-300 in young animals (Fig. 2C and D). Similar to young rats, the application of LFS-300 failed to induce stable LTD in vehicle-injected middle-aged (9–16 months of age) anaesthetized animals (Fig. 2E and F). Remarkably, in middle-aged rats, the lower volume (6 µL) of the ‘AD1’ extract, immunodepleted by anti-Aβ polyclonal antibody AW7, facilitated a robust and stable LTD (Fig. 2G and H). Significantly, immunodepletion of tau with the Tau5 mAb abrogated the LTD facilitation, while mock-immunodepleted with a control mAb 46-4 (AD1/46-4) did not (Fig. 2G and H), thus confirming that this facilitatory effect in middle-aged rats was indeed mediated by tau. Due to the limited amount of the sample, we did not test ‘AD2’ in middle-aged rats.

### Synaptotoxic tau in Ts21 secretomes facilitates LTD in an age-dependent manner

Because synaptotoxic tau in Alzheimer’s disease brain aqueous extracts prepared by homogenization is mostly of intracellular origin, it is important to assess the effect of i.c.v. administration of extracellular synaptotoxic tau on LTD. Previously, we reported that tau in the secretomes of iPSC-derived neurons from people with Ts21, the most common genetic cause of Alzheimer’s disease, inhibited hippocampal LTP *in vivo*.^39^ We wondered if this extracellular source of synaptotoxic tau might also facilitate LTD in an age-dependent manner. Therefore, we tested the effect of Ts21 secretomes on LTD in both young and middle-aged rats. After i.c.v. injection of 10 µL, a volume that inhibits LTP *in vivo*,^39^ of a Ts21 secretome that had been immunodepleted of Aβ, the application of LFS-300 facilitated a very small LTD in young rats (Fig. 3A and B). It is likely that the apparent low potency of these samples is due to the relatively low concentration of tau compared with brain extracts, although we did not increase the volume above 10 µL to test the efficacy of higher doses of tau in young adult rats. However, the same volume of this secretome facilitated the induction of a robust and stable LTD in middle-aged rats (Fig. 3C and D). Immunodepletion of tau with the mAb Tau5 abrogated the LTD facilitation (Fig. 3E and F). We conclude that synaptotoxic tau in Alzheimer’s disease brain and a human iPSC Alzheimer’s disease model facilitates LTD in an age-dependent manner.

### Pick’s disease brain-derived synaptotoxic tau enhances hippocampal LTD

To avoid the concomitant effects on LTD facilitation by both synaptotoxic tau and Aβ in Alzheimer’s disease brain extracts, we further tested brain aqueous extracts from a different tauopathy Pick’s disease. Extracts of PiD1 brain had previously been reported to contain synaptotoxic tau that triggered neurite retraction in cultured human neurons^57^ and inhibited hippocampal LTP *in vitro*^57^ and *in vivo*.^35^ We first injected 10 µL of mock-immunodepleted PiD1 extract, a volume lacking effect on baseline synaptic transmission, into the cerebral ventricles of five young animals, application of LFS-300 induced robust and lasting LTD (Fig. 4A and B). However, when we injected 10 µL of PiD1 extract that was depleted of tau with Tau5 antibody, the LTD enhancement was abrogated (Fig. 4A and B). These findings, combined with the LTD-enhancing effects of Alzheimer’s disease brain aqueous extracts (AD1, AD2) and Ts21 secretomes, confirm our hypothesis that patient brain-derived synaptotoxic tau facilitates hippocampal synaptic LTD.

### Age dependence of the facilitation of LTD by Aβ

Previously, we reported that synthetic Aβ or synaptotoxic Aβ-containing Alzheimer’s disease brain extracts facilitated hippocampal LTD *in vivo*.^10^ Because we found that LTD facilitation by Alzheimer’s disease-related tau was age-dependent and LTP inhibition by synthetic Aβ is increased in middle-aged rats,^41^ we wondered if synaptotoxic Aβ might also facilitate LTD in an age-dependent manner.

Since the most disease-relevant forms of Aβ are present in the brains of patients with Alzheimer’s disease, we first tested the effect of an aqueous extract of Alzheimer’s disease brain (‘AD7’) containing LTP-disrupting Aβ species^35^ on LTD in young rats. Intracerebroventricular injection of 10 µL of Aβ-containing extract, a volume that inhibits LTP *in vivo*,^35^ enabled the weak LFS protocol to induce LTD whereas half this volume (5 µL) was without effect (Fig. 5A and B). Nevertheless, this lower volume (5 µL) of Aβ-containing extract was sufficient to enhance LTD in middle-aged rats (Fig. 5C and D). Importantly, Aβ-immunodepleted AD7 extract failed to facilitate LTD in young (Fig. 5A and B) or middle-aged rats (Fig. 5C and D) whereas co-injection of the anti-tau antibody Tau5 did not affect the facilitation of LTD by Aβ-containing AD7 extract (Fig. 5C and D). These results indicate that AD7 facilitates LTD in an Aβ-dependent manner. Consistent with an age-dependent facilitation of LTD by Aβ-containing extract, synthetic Aβ also facilitated LTD in an age-dependent manner (Fig. 5E and F). In young animals, i.c.v. injection of synthetic oligomeric Aβ enabled the induction of LTD by the weak LFS protocol when using a dose of 585 pmol (5 µL) (Fig. 5E and F) that inhibits LTP.^58^ Half this Aβ dose (292.5 pmol, 2.5 µL) failed to enable LTD by weak LFS in young rats but this lower dose was sufficient to facilitate LTD in middle-aged rats (Fig. 5E and F). Taken together, these data provide strong evidence that ageing enhances the synaptic plasticity-disrupting action of Aβ.

### A TNF-α inhibitor, etanercept, ameliorates both tau- and Aβ-facilitated LTD in middle-aged animals

Our findings that both Alzheimer’s disease-related synaptotoxic tau and Aβ facilitated LTD in an age-dependent manner implicate a shared synaptic depression-promoting action of Aβ and tau in early Alzheimer’s disease pathogenesis. Both Aβ and ageing trigger an increase in pro-inflammatory cytokines, including the master regulator of inflammation TNF-α,^46^ and TNF-α is known to mediate the inhibition of LTP by Aβ^59-61^ and ageing.^62^ Furthermore, tau triggers pro-inflammatory states, which are associated with increased TNF-α levels.^63,64^ Because TNF-α can promote certain forms of LTD, via activation of TNFR1,^59,65^ we hypothesized that TNF-α would be required for Alzheimer’s disease brain tau- and Aβ-facilitated LTD. We tested the role of this cytokine in middle-aged rats using etanercept, which is a decoy receptor for TNF-α.^50^ In middle-aged rats, co-injection of etanercept (50 µg in 5 µL, i.c.v.)^52^ significantly reduced the induction of LTD by LFS-300 in the presence of Alzheimer’s disease brain tau (Fig. 6A and B). Similarly, in middle-aged rats, neither synthetic nor human/patient brain-derived Aβ facilitated LTD in the presence of etanercept (Fig. 6C and D), leading us to conclude that TNF-α could serve as a critical mediator of LTD-facilitating effects of tau and Aβ present in Alzheimer’s disease brain aqueous extracts. However, the failure to fully prevent tau-mediated LTD facilitation by etanercept indicates the possibility that other molecular mechanisms are also involved in Alzheimer’s disease-related tau-enhanced LTD.

### Systemic administration of ISRIB prevents the facilitation of LTD by tau and Aβ in middle-aged animals

Pro-inflammatory stimuli can trigger cellular stress,^43,47,66,67^ which in turn activates numerous cellular pathways, including the integrated stress response (ISR).^47^ The ISR initiates a decrease in general protein synthesis,^68^ which is necessary for many forms of long-lasting synaptic plasticity and memory.^47,68^ Indeed, reducing the ISR facilitates certain forms of LTP and memory in Aβ-overexpressing^69-71^ and aged^54^ mice. Having found that a brain-penetrant, small molecule inhibitor of the ISR, trans-ISRIB,^51^ prevents Aβ-facilitated LTD and abrogates spatial learning and memory deficits in rats injected with synthetic Aβ,^53^ we systemically administered ISRIB (2.5 mg/kg, i.p.) 24 h prior to i.c.v. injection of Alzheimer’s disease brain tau in middle-aged rats. Consistent with the ISR playing an essential role, ISRIB completely abrogated the facilitation of LTD by Alzheimer’s disease brain tau (Fig. 7A and B). Having found that Alzheimer’s disease brain tau-mediated LTD facilitation required the ISR, next we assessed its role in the facilitatory action of Alzheimer’s disease brain Aβ. Similarly, pre-treatment with ISRIB completely prevented the facilitation of LTD by Alzheimer’s disease brain Aβ (Fig. 7C and D). We conclude that the facilitation of LTD by Alzheimer’s disease brain tau and Aβ share similar mechanisms.

## Discussion

We report here that patient-derived tau, like Aβ, can facilitate the induction of synaptic LTD. Furthermore, the facilitation of LTD by tau and Aβ was age-dependent, such that doses of either protein that did not affect LTD in young rats, lowered the threshold for LTD induction in middle-aged animals. Consistent with a role for pro-inflammatory cytokines and protein synthesis disruption, both tau- and Aβ-facilitated LTD were prevented by agents inhibiting TNF-α and the integrated stress response.

Our new data, that diffusible tau obtained from Alzheimer’s disease brain facilitated the induction of LTD by LFS, support an extended synaptic weakening hypothesis for synaptic failure in early Alzheimer’s disease. Whereas previous proposals had restricted themselves to a role for certain synaptotoxic forms of Aβ in driving synaptic weakening, the present work, taken in combination with research reporting LTP inhibitory actions of Alzheimer’s disease brain tau,^34,39,72,73^ helps establish tau as a key player as well. Very recently, we reported the interaction of Aβ and tau synaptotoxicity, with relatively low volumes of certain Alzheimer’s disease brain extracts inhibiting LTP in a manner that requires the presence of both Aβ and tau.^35^ Whether or not this interaction affects LTD facilitation by Aβ and tau in Alzheimer’s disease brain extracts remains to be determined. Indeed, a number of authors have suggested that the impairment of LTP and promotion of LTD mechanisms are likely two sides of the same coin enabling synaptic pruning, with long-lasting structural plasticity including synaptic loss.^3,74-77^

Aqueous brain extracts contain multiple forms of tau derived from both intracellular and extracellular sources.^78,79^ Although tau is primarily an intracellular protein where detergent insoluble fibrils are deposited as neurofibrillary tangles, potentially synaptotoxic forms of tau are released extracellularly.^38,79-84^ For example, an extracellular, Tau5-binding form of tau secreted by Ts21 iPSC-derived neurons potently inhibits LTP^39^ and facilitates LTD (this study). These secretomes contain an array of tau fragments with little or no detectable full-length protein.^39^ Therapeutic targeting of different forms of extracellular tau with selective antibodies has become a major area of clinical development in the potential treatment of Alzheimer’s disease.^85,86^ It is a matter of some controversy whether to target the N-terminus, mid-region or C-terminus of tau. Our finding that a mid-region-directed anti-tau antibody abrogates the LTD facilitation by patient-derived tau lends support to these strategies.^87^ Future studies should further determine the relative efficacy of targeting different tau species on synaptic plasticity disruption by patient-derived tau.

Whereas tau in Alzheimer’s disease and Pick’s disease brain aqueous extracts, as well as Ts21 secretomes, facilitate LTD, relatively soluble tau aggregates prepared by sonication of recombinant tau fibrils were found to inhibit, rather than facilitate, LTD.^88^ Interestingly, tau oligomers in Alzheimer’s disease brain have been reported to have a relatively low molecular weight.^36-38^ Very low amounts of full-length tau, but abundant mid-region fragments and lower levels of N-terminal and C-terminal fragments, are found in the Ts21 iPSC secretomes, which inhibit hippocampal LTP^39^ and facilitated LTD in the present study. Future studies will be needed to establish which isoforms and species (e.g. aggregation, truncation and phosphorylation status) of tau mediate the potent facilitation of LTD by tau found in Ts21 iPSC secretomes and Alzheimer’s disease brain extracts.

The finding that the facilitation of LTD by synthetic Aβ is enhanced in middle-aged rats complements a previous report that the threshold for the inhibition of LTP by synthetic Aβ is lowered in similarly aged animals.^41^ The discovery that both Alzheimer’s disease brain Aβ- and tau-mediated LTD facilitation were also enhanced in an age-dependent manner lends further support to the potential importance of synaptic weakening processes in mediating early Alzheimer’s disease.^12-15,89^ This finding is consistent with evidence in humans that changes in Aβ and tau fluid markers of Alzheimer’s disease commence in mid-life,^90^ usually decades before the onset and clinical diagnosis of dementia. Although the present findings leave open the question as to the mechanisms of how the threshold for Aβ- and tau-induced synaptotoxicity is lowered in middle-aged rats, they help explain why ageing is a primary risk factor and support the development of interventions that target Aβ and tau in middle age prior to presentation with full-blown clinical dementia in older life.

Diffusible forms of Alzheimer’s disease Aβ have been shown to induce hyperexcitability in individual neurons and neural circuits via disrupting excitatory/inhibitory balance.^91^ These mechanisms may underlie the facilitation of hippocampal LTD by Aβ.^15,32^ In the case of tau, epileptogenic hyperexcitability has been found in animal models including Tau4RTg2652 mice exhibiting ∼12-fold overexpression of human tau relative to endogenous mouse tau,^92^ transgenic mice with neuronal expression of A152T-variant human tau (hTau-A152T)^93^ and FTDP-17 (frontotemporal dementia with parkinsonism linked to chromosome 17) mice overexpressing a human tau isoform with two N-terminal inserts, four-microtubule-binding-repeat elements and three FTDP-17-linked mutations G272 V, P301L and R406W under the control of the neuron-specific Thy-1 promoter (human Tau^VLW^).^94^ Whether, like Aβ, tau also causes hyperexcitability via excitatory/inhibitory imbalance, and if so, which variant of tau, remains elusive.^87^

It is remarkable that apart from sharing the ability to facilitate LTD in a similar age-dependent manner, both Aβ and tau require TNF-α and the ISR to disrupt this form of plasticity. Our working hypothesis was based on previous reports that Aβ increases TNF-α in the brain^58,95-97^ and thereby drives the ISR excessively.^66^ Furthermore, etanercept and related TNF-α-neutralizing strategies prevent Aβ-mediated activation of the ISR^66^ and inhibition of LTP,^60,98^ and suppression of the ISR also prevents Aβ-mediated LTP inhibition.^69-71^ Indeed, both TNF-α activation of TNFR1^59,65^ and other activators of the ISR^53,99-102^ can promote certain forms of LTD. Less is known about how tau affects TNF-α,^103^ but tau triggers pro-inflammatory states, which are associated with increased TNF-α levels.^63,64^ The ISR is also engaged in certain models of tauopathy^104-106^ but not others.^107,108^ The ISR is a complex intracellular signalling pathway that helps the cell, tissue and organism to adapt to variable intrinsic and extrinsic stress. Given that synaptotoxic Aβ and tau species induce ISR hyperactivation, suppression of the ISR by ISRIB could mediate its beneficial effects against the synaptic plasticity disruptive actions of both Aβ and tau. Although the safety of ISRIB in humans has yet to be evaluated, systemic administration of ISRIB restores translation downstream of kinase phosphorylation of the eukaryotic initiation factor 2 α-subunit (eIF2α), without obvious behavioural side effects at the dose used in the present experiments.^53^ ISRIB blocks metabotropic glutamate receptor (mGluR)-dependent LTD induced by the mGluR agonist DHPG^99^ and Aβ-mediated mGluR-LTD,^53^ while it does not observably affect control LTD induced by LFS-900 in live rats.^109^ Direct activation of the ISR by synaptotoxic Aβ and tau species may also drive TNF-α production, possibly in a positive feedback loop. Normal concentrations of TNF-α can directly affect neurons expressing TNF-α receptors, impacting basal synaptic transmission and synaptic plasticity by affecting NMDARs or AMPARs.^110^ At higher concentration, such as that found in Alzheimer’s disease brain, TNF-α also signals to astrocytes expressing TNFR1. The activation of TNFR1 in astrocytes in the hippocampus triggers the release of glutamate.^111-114^ The astrocyte-released glutamate enhances presynaptic glutamate release via activating presynaptic GluN2B-containing NMDAR.^114^ The activation of NMDARs, both synaptic and extrasynaptic, is critical for the induction of many forms of LTP and LTD.^115^ In Aβ Alzheimer’s disease models, extrasynaptic glutamate receptors, in particular, GluN2B and mGluR5, are responsible for LTP impairment^60,116^ and LTD enhancement.^5,9-113^ TNF-α has been found to mediate LTP impairment in different animal models of Alzheimer’s disease.^59,98,117^ Having found Aβ-mediated facilitation of mGluR5-LTD^10,11,53^ and the involvement of TNFR1 in the induction of mGluR5-LTD,^118^ we report here that anti-TNF-α agent prevents LTD facilitation mediated by both Aβ and tau.

Inflammaging, a chronic low-grade inflammation in the body caused by accumulated oxidative stress and cellular senescence events,^119^ leads to age-associated pro-inflammatory changes in the brain,^120^ including increased TNF-α. Such changes may contribute to the lowered threshold for LTD facilitation by both Aβ and tau in middle-aged rats in the present study. Whether inflammaging is involved in the age-dependent LTD facilitation or whether ISR-inhibition and TNF-α-neutralization provide beneficial effects to ameliorate inflammaging needs to be carefully investigated.

The finding that diffusible forms of tau and Aβ species in Alzheimer’s disease brain both promote LTD via shared downstream mechanisms lends support to further exploring the synaptic weakening hypothesis of Alzheimer’s disease and detailed underlying cellular processes. In addition to the interplay between pro-inflammatory processes and the ISR, other shared mechanisms for Aβ and tau, such as cellular prion protein (PrP^C^) and low-density lipoprotein receptor-related protein 1 (LRP1), have been reported. Indeed, we recently discovered that PrP^C^ plays a pivotal role in the synaptotoxicity mediated by soluble Aβ, tau and α-synuclein.^57^ Furthermore, PrP^C^ is implicated in Aβ-facilitated LTD^10^ and in the impairment of LTP by both Aβ and tau.^34,39^ LRP1 is known to regulate the uptake of both Aβ^121^ and tau.^122^ The potential involvement of these and other putative shared mechanisms in synaptic weakening by Aβ and tau warrants future investigation.

## Data Availability

All data supporting this study are available from the corresponding author upon reasonable request.
